# Surgical Outcomes of a Human Umbilical Cord Allograft Over the Ahmed Glaucoma Valve Plate for Refractory Glaucoma

**DOI:** 10.7759/cureus.67923

**Published:** 2024-08-27

**Authors:** Aparna Rao

**Affiliations:** 1 Glaucoma, L V Prasad Eye Institute - Kallam Anji Reddy Campus, Hyderabad, IND

**Keywords:** allograft, fibrosis, wound healing, umbilical cord, ahmed glaucoma valve

## Abstract

Objectives: To report the surgical outcomes of sterile dehydrated human umbilical cord allograft over the Ahmed glaucoma valve (AGV) plate for refractory glaucoma.

Methods: Thirty-four eyes of 34 patients with refractory glaucoma who underwent AGV with umbilical cord allograft (AmnioPlast THICK™; Life Cell International Private Limited, Mumbai, India) placed over the AGV plate between September 2021 and 2022 at a tertiary eye care centre were included (group1) and compared with 30 eyes of 30 patients undergoing AGV without amnioblasts (group 2). The intraocular pressure (IOP) at day one, one month, six months, and final IOP; the necessity for medications; or additional surgeries for IOP control were extracted from the hospital database. Success was defined as achieving an IOP below 22 mm Hg with or without glaucoma medications. IOP spikes after surgery were identified as a rise in pressure beyond 22 mm Hg at any point beyond six weeks post-surgery following an initial reduction of pressure exceeding 30% from the baseline pre-surgical IOP.

Results: A notable reduction in intraocular pressure (37 ± 7.9 mm Hg preoperative versus 14 ± 3.7 mm Hg at the final follow-up and 28 ± 3.6 mm Hg versus 18 ± 6.7 mm Hg in group 1 and 2, respectively) was observed in all eyes, with successful outcomes observed in 23 out of 34 eyes (67%). Ten eyes experienced a pressure spike, occurring at a median time of 12 months (range: 6-18 months), predominantly beyond six months post-surgery in group 1, while group 2 had similar pressure spikes in 21 of 30 eyes at a median time of two months (range: 1-5 months). No eyes necessitated supplementary glaucoma procedures, concluding with a final IOP of 14 ± 3.7 mm Hg at 1.9 ± 0.8 years. Vision loss occurred in only one of the 11 eyes that failed owing to non-glaucoma-related causes in group 1.

Conclusions: The human umbilical cord allograft plate over the AGV plate may help in postponing the onset of intraocular pressure spikes beyond traditionally defined timelines. This helps in reducing and delaying the hypertensive phase occurring due to fibrosis.

## Introduction

The Ahmed glaucoma valve (AGV) has brought about a significant transformation in surgical results for challenging cases of glaucoma that are at a heightened risk of failure following standard trabeculectomy [1-5]. This breakthrough has empowered surgeons to rescue and preserve vision in numerous refractory glaucoma cases potentially, encompassing conditions such as neovascular glaucoma, uveitis glaucoma, and instances following unsuccessful trabeculectomy [3-5]. It represents a valuable advancement in the prevention of blindness in these particularly high-risk eyes. Landmark studies have reported a fair five-year intraocular pressure (IOP) outcome after AGV or valved implants, with the failure rates being higher for valved implants such as AGV [3,4]. The excessive fibrous reaction to foreign materials remains a significant worry associated with these implants, potentially causing a hypertensive phase or implant failure following AGV surgery [4,6-8]. This impacts the overall success rates in terms of IOP control and surgical outcomes after AGV, especially in refractory glaucoma. While procedures such as needling, anti-mitotic application, and excision of the fibrous capsule over the plate have been outlined for addressing problematic blebs after AGV, these methods are marked by a notable risk of implant protrusion, wound separation, and treatment inefficacy [3,4,6,9,10]. Employing less invasive strategies to diminish the fibrotic response may serve as a viable means to reduce the necessity for AGV revisions or to achieve an optimal surgical outcome in refractory glaucoma.

There have been some innovative uses and potential applications for the umbilical cord and its components [11,12]. Research suggests that umbilical cord tissue can be processed and used in wound healing and tissue repair, particularly in cases of non-healing wounds. These tissues can contain growth factors and stem cells that promote tissue regeneration. Intuitively, this may also be useful to mitigate the fibrotic response after AGV incited over the AGV plate and may improve the surgical outcomes in refractory glaucoma. This series reports the clinical efficacy of the umbilical cord allograft with AGV compared to AGV only for refractory glaucoma.

## Materials and methods

All cases of refractory glaucoma that underwent AGV with or without allograft placed over the plate by a single surgeon (APR) between September 2020 and September 2023 at a tertiary eye center were compared for this retrospective study (the commercially available sterile dehydrated human umbilical cord allograft was available for our use only from 2021). The study was approved by the institutional review board (IEC-16-IM-3), and informed written consent was taken from all patients. Refractory glaucoma included those that had failed trabeculectomy for uncontrolled IOP despite maximal medical therapy status post-retinal or keratoplasty/refractive surgeries, presence of severe peripheral anterior synechiae with shallow anterior chamber (as seen in secondary developmental glaucoma), or conjunctival scarring. Those with a minimum follow-up of six months after AGV were selected for the final analysis.

All surgeries were done in the standard manner described elsewhere [1-3]. Briefly, AGV surgery was performed by a fornix-based superotemporal conjunctival flap and using the AGV model (FP7; New World Medical, Rancho Cucamonga, CA). The plate was fixed using a 10-0 prolene suture 8 mm from the limbus. The tube was inserted 2-3 mm in the anterior chamber or sulcus after trimming and was secured with a 10-0 nylon suture. The tube was inserted through a 24-gauge needle scleral track into the anterior chamber after priming with 1-2 mL of balanced salt solution and trimming to ensure that 1.5-2 mm was placed in the anterior chamber.

**Figure 1:**
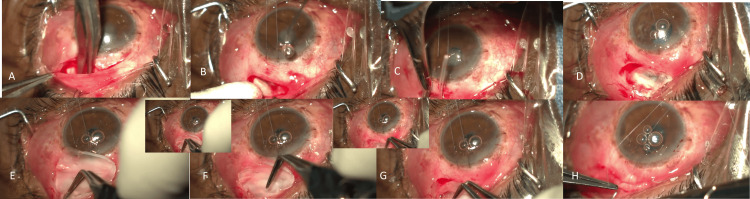
Intraoperative steps of the umbilical cord allograft with Ahmed glaucoma valve surgery A-H shows the procedure of inserting and sliding a commercially available sterile human umbilical cord allograft over the Ahmed glaucoma valve plate in the superotemporal pocket in a patient with neovascular glaucoma. E-H with insets shows the steps of gradually sliding the allograft over the plate after plate fixation.

For the eyes in group 1, the AGV plate was now covered with a commercially available sterile dehydrated human umbilical cord allograft (AmnioPlast THICK™; Life Cell International Private Limited, Mumbai, India), sized 5x5 mm after wetting with sterile balanced salt solution, and making sure that it was resized to completely cover the plate in the conjunctival pocket (Figure 1). The allograft was slid over the plate after plate fixation by smooth non-toothed forceps, with no fixation sutures or glue being used. The allograft being flexible, slides easily into the superotemporal pocket over the AGV plate.

Further steps of the surgery were common for groups 1 and 2. The tube was fixed using 10-0 prolene sutures and was covered with a partial thickness scleral patch graft (prepared from rejected corneas available, which were deemed unsuitable for penetrating keratoplasty by the hospital eye bank repository), which was secured using fibrin glue. The conjunctiva was closed using continuous 8-0 Vicryl sutures. Postoperatively, the allograft was not visible during the healing phase possibly gradually undergoing gradual integration with the capsule over the plate.

Postoperatively, all patients were initiated on prednisolone acetate 1% gradually tapered over four weeks. Postoperative visits were scheduled on day one, day one, one month, and thereafter every three months. Patients were referred to retina services for appropriate retinal intervention as and when required for PRP, additional bevacizumab injections, or other treatments after dilated fundus evaluation at each visit from one month postoperatively. The number of retinal interventions post-surgery was noted. Any displacement of the allograft or any infection in the postoperative period was also noted.

Details that were retrieved from the hospital database included the presenting IOP before surgery, IOP at one day, one month, six months, and final follow-up; diagnosis; demographic details; best corrected visual acuity; need for medications after surgery; complications; need for repeat surgeries; and final IOP at the last visit.

Success was defined as IOP <22 mm Hg with or without glaucoma medications at six months (both qualified and complete success were considered success since a hypertensive defined below is a natural course of the implant healing response and does not signify failure as for routine filtering surgeries), with failure defined as IOP > 22 despite maximum medical treatment on two consecutive follow-up visits after three to six months, loss of vision, or the need for additional surgery to control IOP (needling, bleb revision, wound repair). The hypertensive phase was defined as the development of raised IOP > 22 mm Hg at any time two weeks after surgery, after an initial reduction of IOP > 30% from the pre-surgical IOP. The need for retinal intervention or the need for antiglaucoma medication for controlling the hypertensive phase was not classified as failure unless the basic disease for neovascular glaucoma (NVG) such as proliferative diabetic retinopathy and retinal vein occlusion) had not progressed. Data were collected to identify the time of onset of the hypertensive phase after surgery and the final outcomes in terms of additional procedures required in that eye. The final IOP outcomes, visual outcomes, need for medications after surgery, and the rate of hypertensive phase or failure were analyzed.

Statistics

All analysis was done using the Stata version (version 12; Stata Corp, College Station, TX). Continuous data are presented as mean ± SD or median (range). Clinical variables between the two groups were compared using an unpaired student T-test or Wilcoxon-Mann-Whitney test (for non-parametric data). The success rates and failure rates were analyzed with the reasons for failure noted.

## Results

We included 34 eyes of 34 patients in group 1 and 30 eyes of 30 patients in group 2 (males: females 27:7 and 23:7 in groups 1 and 2, respectively) with a mean higher IOP at surgery in group 1 that was not statistically significant (Table 1). Most patients (83%) were on > 3 medications in both groups (Table 1).

**Table 1:** Clinical and demographic profile of patients undergoing Ahmed glaucoma valve surgery with dehydrated human umbilical cord allograft IOP-Intraocular pressure, VR-Vitreoretinal, ICE-Iridocorneoendothelial syndrome, NVG-neovascular glaucoma, *-see text for the definition of success

Variable (n=34)	Group1 (n=34) Mean±Standard deviation. or N (%)	Group 2 (n=30) Mean±Standard deviation. or N(%)	P value
Age (years)	47±17.3	38±12.4	0.06
IOP before surgery (mm Hg)	37±7.9	28±3.6	0.054
NVG	14 (41.1)	15 (50)	NA
Post VR	12 (35.3)	12 (40)	NA
Post keratoplasty	4 (11.8)	2 (6.7)	NA
ICE syndrome	3 (8.9)	1 (3.3)	NA
Other	1 (2.9)	0 (0)	NA
3 topical+/-systemic medications	28 (82.5)	16 (53.3)	0.051
4 topical+/-systemic medications	2 (5.8)	4 (13.3)	0.1
>4 topical+/-systemic medications	4 (11.7)	4 (13.3)	0.09
Success*	23 (67)	16 (53.3)	0.2
Time of hypertensive phase (n=10)	10±5.2	5±3.8	0.001
Hypertensive phase <3months	2 (5.8)	18 (60)	0.01
Hypertensive phase >6 months	8 (23.5)	3 (10)	0.04

No intraoperative complications were encountered in any eye in both groups. Postoperatively, minimal hyphema was seen in four eyes with NVG in group 1 and eight eyes in group 2, with one eye having a shallow anterior chamber for one week that resolved spontaneously. Six eyes in group 1 and nine eyes in group 2 also underwent concurrent cataract surgery for significant cataracts, with all cases having intraocular lens implantation and AGV performed at the same sitting. Twelve eyes in group 1 and 13 eyes in group 2 with retinal associations underwent repeat procedures such as add pan-retinal photocoagulation (n=12), cataract extraction with or without vitrectomy/intravitreal injections (n=11), graft suture removal (n=2), and repeat vitrectomy/intravitreal injections/repeat partial silicon oil removal (n=3). One patient was lost to follow-up after advice for retinal photocoagulation two months owing to high uncontrolled blood sugars after vitrectomy and returned with recurrence of NVG and loss of vision. None of the eyes required repeat glaucoma procedures or repeat filtering procedures at the final follow-up.

In group 1, 14 eyes required one to two medications at the final follow-up, with four eyes being prescribed medications outside by local ophthalmologists without the event of an IOP spike noted in any eye. Group 2 eyes required meditations for IOP control though they required it earlier than group 1 with no significant difference in the number of medications at the final follow-up (Table 1). The mean IOP and the number of antiglaucoma medications were reduced in all cases (Table 2). Of 10 eyes developing a delayed IOP spike, seven cases developed it at > 6 months, with three developing at two to six months. Group 2 eyes had an IOP spike earlier than group 1 with 18 of 30 eyes having a spike at <3 months (Tables 1-2).

**Table 2:** Intraocular pressure profile of patients that underwent Ahmed glaucoma valve surgery with a sterile dehydrated human umbilical cord allograft IOP-Intraocular pressure, P values based on unpaired student T-test or non-parametric tests; see text for full description.

Variable	Mean+SD Group 1	Minimum	Maximum	Mean+SD Group 2	Minimum	Maximum	P value
IOP 1 day (mm Hg)	14±5.3	6	21	12±4.9	10	11	0.8
IOP 1 month (mm Hg)	15±5.4	8	20	22±5.8	18	28	0.04
IOP 3 month (mm Hg)	21±9.7	10	32	18±9.8	10	27	0.6
IOP 6 months (mm Hg)	19±5.8	8	26	16±7.6	9	23	0.5
Final IOP (mm Hg)	14.±3.7	8	19	16±5.7	11	21	0.2
Antiglaucoma medications	1.±0.9	0	2	1±1.2	0	2	0.9

The median time for the development of an IOP spike in group 1 was 12 months (range: 2-18 months) and five months (range: 1-8 months) with only two eyes in group 1 developing it at < 3 months (p=0.001, Table 1). Both the eyes in group 1 developed an early IOP spike < 3 months and underwent repeat pars plana vitrectomy (PPV), after which the IOP spike was noted mandating antiglaucoma medications. Of 21 eyes that developed IOP spikes in group 2, 60% presented within three months, with most achieving IOP control with the addition of antiglaucoma medications. One patient in group 1, a known diabetic and on dialysis, was lost to follow-up after PPV defaulting advice for repeat retinal photocoagulation and returned with repeat vitreous hemorrhage and loss of vision owing to advanced proliferative diabetic retinopathy with neovascular glaucoma. Five patients (n=1 in group 1; n=4 in group 2; n=2 with iridocorneal endothelial syndrome (ICE) syndrome; one with NVG; two with post-keratoplasty) developed corneal decompensation with a reduction in vision by two to four Snellen lines of visual acuity after AGV, which mandated keratoplasty. Six patients with NVG in group 1 and seven eyes in group 2 developed repeat vitreous hemorrhage and/or hyphema after cataract surgery after AGV, requiring add PRP, or intravitreal injections/PPV, with one patient that was lost to follow-up after vitrectomy returning with complete loss of vision. Success was seen in 23 of 34 eyes (67.8%) and 16 of 30 (53.3%) eyes in group 2 (Table 1). Failure seen in 11 eyes and 14 eyes in group 2 contributed to reduction/loss of vision by corneal decompensation or repeat vitreous hemorrhage at the final follow-up with none of the eyes needing additional procedures for IOP control.

## Discussion

This study found a success rate of 67.8% in the eyes of group 1 and 53.3% in the eyes of group 2 with refractory glaucoma undergoing AGV with a significantly delayed IOP spike in group 1 with the use of umbilical cord allograft. Failure in both groups owing to reduction of vision by IOP-unrelated causes (loss of vision in one eye), with none of the eyes requiring additional glaucoma procedures for IOP control.

We observed a delayed IOP rise with the use of AGV with umbilical cord allograft over the plate, which we did not classify as hypertensive phase, owing to the significant delay in the onset of the period of IOP spike seen in this study and difficulty in ascertaining the difference between a delayed hypertensive phase and failure. However, the delayed IOP spike suggests a delayed hypertensive phase owing to a reduction in the fibrotic capsule formation over the pate after the use of umbilical cord allograft in group 1. An IOP rise that is seen at two to six months after AGV is traditionally called a hypertensive phase and is attributed to an exuberant fibrotic response. Though this occurs over a wide range of periods after surgery, the incidence varies ranging from 31%-57% across studies [6-10]. In contrast, failure is an immediate postoperative IOP spike that occurs immediately or within six months after surgery. Studies have identified a higher pre-operative IOP and younger age as risk factors for developing a hypertensive phase, which can lead to failure in some eyes [6-8]. Various attempts have been made to reduce the hypertensive phase, including early aqueous suppression, biodegradable collagen matrix, use of a pediatric implant with reduced plate size, amniotic membrane, mitomycin-C, and anterior chamber washout [13-20]. While aqueous suppression is reported to reduce the incidence of the hypertensive phase, this however does not impact the overall long-term success or failure rates or the final IOP [13,15,16,20]. While studies have reported the use of antimitotic or excision of the fibrous capsule, literature on the use of allografts or other modulating grafts over the AGV plate to reduce fibrosis is non-existing [13-15,18,19]. We believe that the umbilical cord allograft placed over the AGV plate reduces the fibrotic response directly at that site thereby delaying the onset of IOP spikes causing hypertensive phase. This needs further validation with anterior segment imaging to outline differences between a traditional and a mitigated delayed hypertensive response after AGV.

The commercially available sterile dehydrated human umbilical cord allograft has been used for treating the ocular wound and has potential use as a patch graft over the tube after AGV [11,12,21,22]. While the cord lining provides a protective barrier, the Wharton jelly and extracellular matrix component of the allograft act as a biocompatible scaffold and biological barrier to fibrosis [11,21,22]. We, therefore, chose to use it to cover the pate to reduce the wound healing responses in refractory glaucoma in this study. Ameliorating the encapsulation of the AGV plate can be beneficial in reducing the fibrotic response in the eye following AGV surgery, consequently preventing the hypertensive phase or failure after AGV. Long-term success rates after AGV surgery have been reported to vary between 60% and 85% in various studies, including the TVT and ABC studies [1,3,4]. Although risk factors such as NVG, higher baseline IOP, and lower visual acuity at the time of presentation have been identified as potential contributors to AGV failure, this study revealed comparable success rates of 67% with an IOP spike delayed > 2 months unlike traditional hypertensive phase after plain AGV after the addition of an umbilical cord allograft over the AGV plate in cases of refractory glaucoma, including NVG [3,4]. Notably, the incidence of the IOP spike was lower and occurred later, a result that could potentially be attributed to the antifibrotic activity of the umbilical cord allograft at the plate site, which may aid in reducing the wound healing response and the IOP outcomes after AGV in refractory glaucoma.

The use of umbilical cord or its derived products in wound healing is an emerging and promising field of regenerative medicine [11,12,21,22]. Umbilical cord tissue contains various components that can facilitate wound healing and tissue repair. It has anti-inflammatory and immunomodulatory effects that can help control the body's immune response at the wound site, reducing excessive inflammation and promoting an environment conducive to healing [12,21,22]. In the eye, mesenchymal stem cells derived from the umbilical cord or its derived eye drops have been evaluated in murine or in-vitro experimental models of corneal epithelial injury in promoting corneal wound healing or corneal endothelial regeneration [21,22]. This study showed its potential use in reducing the IOP spike owing to fibrosis after AGV in refractory glaucoma. Further, the mechanism by which the IOP spike after AGV is ameliorated needs further in-depth study using in-vitro or animal models.

Limitations

This pilot study had several limitations apart from its retrospective design and small sample size. Since we wanted to evaluate the clinical utility of the allograft placed over the AGV plate, which is a novel way of reducing fibrosis, we did not design a randomized trial using other techniques such as aqueous suppression or adjuvant mitomycin-C. We also included only refractory glaucoma with a high risk of failure and did not use bleb imaging techniques to study the tissue characteristics after AGV. Nevertheless, the study shows a potential use of cord allografts for reducing the wound healing response after AGV, thereby mitigating or delaying the hypertensive phase. The use of this allograft can be expanded to other indications in glaucoma surgical practice in future studies.

## Conclusions

There is a compelling need for devising methods to improve surgical outcomes after the use of drainage devices in refractory glaucoma. The fibrosis that occurs over the plate of the valve is a major contributor to the development of a hypertensive phase and an IOP spike after surgery. Several methods have been tried to reduce the onset of the hypertensive phase or delay the onset. An exuberant fibrotic response in these situations can also cause failure after drainage devices in refractory glaucoma. This study highlights the importance of the efficacy of dehydrated umbilical cord allograft over the pate to reduce the fibrotic response and delay the onset of the IOP spike that heralds the development of the hypertensive phase. This can help prevent the number of such spikes and delay the onset of spikes, thereby improving the long-term outcomes in refractory glaucoma.
